# Gut microbiota and butyrate contribute to nonalcoholic fatty liver disease in premenopause due to estrogen deficiency

**DOI:** 10.1371/journal.pone.0262855

**Published:** 2022-02-02

**Authors:** Limin Liu, Qingsong Fu, Tiantian Li, Kai Shao, Xing Zhu, Yingzi Cong, Xiaoyun Zhao

**Affiliations:** 1 Department of Medical Experiment Center, Qilu Hospital (Qingdao), Cheeloo College of Medicine, Shandong University, Qingdao, China; 2 Department of Qingdao Key Lab of Mitochondrial Medicine, Qilu Hospital (Qingdao), Cheeloo College of Medicine, Shandong University, Qingdao, China; 3 Department of Pathology, Qilu Hospital (Qingdao), Cheeloo College of Medicine, Shandong University, Qingdao, China; 4 Department of Microbiology and Immunology, University of Texas Medical Branch, Galveston, TX, United States of America; 5 Department of Pathology, University of Texas Medical Branch, Galveston, TX, United States of America; Texas A&M University, UNITED STATES

## Abstract

The incidence of nonalcoholic fatty liver disease (NAFLD) in postmenopausal women has increased significantly. Estrogen plays a very important role in NAFLD, but whether NAFLD in premenopausal women was caused by estrogen deficiency was unknown. Thus, it is of great clinical significance to explore the mechanism of NAFLD in premenopausal women. Gut microbiota and its metabolites short chain fatty acids (SCFA) have been shown to play important roles in the development of NAFLD. In this study, we investigated the impact of gut microbiota and SCFA in NAFLD patients and mice caused by estrogen deficiency. We showed that premenopause NAFLD patients had much lower estrogen levels. Estrogen deficient mice, due to ovariectomy (OVX), suffered more severe liver steatosis with an elevated body weight, abdominal fat weight, serum triglycerides and deterioration in hepatic steatosis. Altered gut microbiota composition and decreased butyrate content were found in NAFLD patients and in OVX mice. Furthermore, fecal microbiota transplantation (FMT) or supplementing with butyrate alleviated NAFLD in OVX mice. The production of antimicrobial peptides (AMP) Reg3ɣ, β-defensins and the expression of intestinal epithelial tight junction, including ZO-1 and Occluding-5, were decreased in the OVX mice compared to control mice. Upregulation of PPAR-ɣ and VLDLR, downregulation of PPAR-ɑ indicated that OVX mice suffered from abnormal lipid metabolism. These data indicate that changes in the gut microbiota and SCFA caused by estrogen reduction, together with a disorder in AMP production and lipid metabolism, promote NAFLD, thus provide SCFAs derived from microbiota as new therapeutic targets for the clinical prevention and treatment of NAFLD.

## 1. Introduction

NAFLD has been recognized as one of the most prevalent etiologies of chronic liver disease worldwide [1]. NAFLD is mainly characterized by fat accumulation in the hepatocytes. Such excessive lipid accumulation can evolve into non-alcoholic steatohepatitis (NASH), cirrhosis, and, finally, hepatocellular carcinoma (HCC) [2]. Thus, patients with NAFLD have a high risk of progressing to cirrhosis and HCC and are at an increased risk for liver-related mortality [3]. Given the great threat posed by NAFLD to human health and its poor prevention strategy, there is a need to characterize its pathogenesis better and to identify potential therapeutic targets for patients with NAFLD.

NAFLD can be effectively inhibited by estrogen. Compared to men, women are generally more resistant to obesity and the accompanying NAFLD, although menopause is a risk factor [4]. NAFLD is found three times more often in women after menopause than before it [5]. It has shown that postmenopausal women who receive hormonal therapy are less likely to develop NAFLD than postmenopausal women who do not [6]. Although NAFLD can be effectively alleviated by estrogen, the severe side effects, such as the increased risk of breast cancer, limit its clinical application. Moreover, the precise mechanisms by which estrogen prevents hepatic steatosis have not been fully elucidated.

An accumulating evidence has indicated that gut microbiota plays an important role in host fat metabolism and in the development of NAFLD [7]. Germ-free mice gained less weight than conventional mice when given a sugar and lipid-rich diet, despite their greater food consumption [8], and developed enhanced susceptibility to hepatic steatosis after restitution with gut microbiota from NAFLD mice [7]. In recent years, further evidence of interaction between the gut and the liver has been emerging. Hepatic products can directly influence the microbiota composition, whereas bacteria may have both direct and indirect effects on liver function and physiology [9, 10], suggesting that the gut-liver axis is an important component in the development of NAFLD. However, it is not known whether the gut microbiota composition is changed in NAFLD induced by estrogen deficiency.

The gut bacterial metabolites also mediate interactions with the host. SCFA are a major class of bacterial metabolites and are mainly produced in the colon by bacterial fermentation of otherwise indigestible fibers [11]. After absorption by the colonocytes, these SCFAs are either used locally as fuel for colonic mucosal epithelial cells or enter the portal bloodstream [12]. Beyond their importance for intestinal integrity, SCFAs have been shown to benefit the host by exerting anti-obesity and anti-diabetic effects [13–15].

The gut microbiota and SCFA associated with NAFLD induced by estrogen deficiency have not yet been systematically assessed. We showed here that the gut microbiota composition and the butyrate content differed between estrogen deficient NAFLD patients/mice and the controls. FMT or supplementation with butyrate can alleviate NAFLD. Furthermore, estrogen deficiency has a potent effect on intestinal epithelial cells and on the lipid metabolism pathway.

## 2. Methods

### 2.1. Patient population

Female NAFLD patients and normal controls, aged between 35–45 years, attending Qilu Hospital of Shandong University between February 2019 and December 2019 were included in our experiment. Fresh feces were centralized collected in December 2019 and frozen immediately at -80°C. We conducted DNA extraction right after the samples collection. Informed consent was signed. We had access to information that could identify individual participants during or after data collection. The diagnostic criteria for NAFLD had to meet the following requirements: 1) No history of drinking, or an ethanol intake of less than 70g/week (female) for at least one year prior to the experiment; 2) Participants were without any of the following: viral hepatitis, drug-induced liver disease, total parenteral nutrition, hepatolenticular degeneration, autoimmune liver disease or any other specific diseases that can lead to fatty liver disease; 3) Liver imaging manifestations conformed to the diagnostic criteria for diffuse fatty liver disease. Patients who had used antibiotics or other special drugs in the past three months for systemic diseases such as diabetes and hypertension and those with unqualified fecal DNA were excluded from participation in the study.

### 2.2. Animals and experimental design

Female C57BL/6 mice aged at 6–8 weeks were purchased from Jinan Pengyue animal company. All animals received humane care following the Guide for the Care and Use of Laboratory Animals approved by Shandong University. Throughout the study period, all animals had access to food and water ad libitum and were maintained on a 12 h light/dark cycle (21 ± 2°C with a relative humidity of 45 ± 5%). After one week of adaptation, animals were randomly divided into the following four groups: normal diet (C group), sham-operated+ high fat diet (HFD, 60 kcal% Fat, research diets, New Brunswick, Canada) (SH group), OVX+HFD (OH group), OVX+HFD+FMT (OHF group). Two weeks after OVX, the mice were given HFD. FMT was performed based on an established protocol [16]. Control animals received the same volume of saline solution. After HFD administration for 4 weeks, mice were anesthetized using 2% Isoflurane followed by cardiac puncture and the blood was collected. Whole livers were immediately prepared for histological analysis; some were stored at -80°C for RNA and western blot preparation, and blood samples were collected for biochemical analysis. Liver function was assayed by the serological activities of alanine aminotransferase (ALT) and aspartate aminotransferase (AST) by using the Hitachi 7600–020 clinical analyzer (Hitachi, Tokyo, Japan).

#### 2.2.1. Statement

We confirm that human and animal experimental protocols were approved by Shandong University ethic licensing committee.

The reference number is KYLL-2019 (KS)-240.

We confirm that informed consent was obtained from all subjects.

### 2.3. Isolation of intestinal epithelial cells (IEC)

The small intestines were incubated with 5 mM EDTA at 37°C for 40 min. Cells were collected by passing the supernatant through a 100-μm cell strainer (BD Falcon, Fremont, CA). After washing with PBS, IEC were separated from a 20/40% Percoll interface (Amersham Pharmacia Biotech, Amersham, UK).

### 2.4. Fecal DNA extraction and 16S rRNA gene sequence analysis of gut microbiota

Fresh feces were collected into individual sterile Ependorf tubes and then frozen immediately at -80°C until DNA extraction. The total bacterial community DNA was extracted using the TIANamp Stool DNA Kit (Tiangen Biotech Co., Ltd., Beijing, China) according to the manufacturer’s instructions.

After DNA extraction from the feces samples, we used polymerase chain reaction (PCR) amplification and pyrosequenced the V3 and V4 regions of the bacterial 16S ribosomal RNA gene (forward primer, 5-ACTCCTACGGGAGGCAGCAG-3; reverse primer, 5-GGACTACHVGGGTWTCTAAT-3). Barcoded V3-V4 amplicons were sequenced using the pair-end method by an Illumina MiSeq sequencing platform at the Shanghai Biotree Biotechnology Company (Shanghai, China). Sequences with an average Phred score lower than 30, containing ambiguous bases, with homopolymer runs exceeding 6 bp, having mismatches in primers, or having a length shorter than 100 bp were removed. Only sequences with an overlap longer than 10 bp and without any mismatches were assembled according to their overlap sequence. Reads that could not be assembled were discarded. Barcode and sequencing primers were trimmed from the assembled sequence [17]. All sequence files are available from the SRA NCBI database (https://www.ncbi.nlm.nih.gov/sra/PRJNA766510).

### 2.5. Histological evaluation

Liver and colon sections (5-μm) were prepared for paraffin-embedded cross-sectioning and stained with hematoxylin and eosin (H&E). Lesion images were captured with a microscope (Olympus, Tokyo, Japan).

### 2.6. Determination of estrogen, insulin and leptin in serum

The levels of estrogen, insulin and leptin in the serum were analyzed with Enzyme-Linked Immunoassay (ELISA) kits (Neobioscience Technology Co., Ltd., Shenzhen, China) according to the manufacturer’s instructions.

### 2.7. Determination of content of SCFAs

According to the reported method [18] with some modifications, the contents of SCFAs including acetic, propionic, n-butyric, i-butyric, n-valeric and i-valeric acids were determined with Agilent 6890 N gas chromatography (GC) equipped with a HP-INNOWAX column (30 m × 0.25 mm × 0.25 m; J & W Scientific, Agilent) and a flame ionization detector (FID). Briefly, the fecal content (50 mg) was mixed with 250 mL of deionized water; 150 mL of the slurry was then mixed with an equal volume of 0.3 mg/mL of 2-ethylbutyric acid (an internal standard). The mixture was centrifuged at 8000 rpm for 5 min, and the supernatant was filtered through a 0.45 m membrane for SCFA analysis. The operating conditions were set as follows: the initial column temperature was kept at 100°C for 1 min, then increased to 180°C at a rate of 5°C /min and kept at 180°C for 4 min. The flow rates for N2, H2, and air makeup gas were set at 30, 30 and 260 mL/min, respectively.

### 2.8. Quantitative real-time PCR

Total RNA was extracted with TRIzol (Life Technologies) and quantified for cDNA synthesis. Quantitative real-time PCR reactions were performed by using SYBR Green Gene Expression Assays (Bio-Rad, Hercules, CA, USA). Quantification was undertaken using the 2^-ΔΔCt^ method (Livak and Schmittgen,2001). All data were normalized to GAPDH mRNA expression.

### 2.9. Immunoblot

Protein concentrations were determined with a bicinchoninic acid (BCA) assay kit (Pierce Biotechnology, Rockford, IL). Primary antibodies against Reg3ϒ, β-defensins 1(Affinity, Cincinnati, USA), Occludin-5, PPAR-ϒ, VLDLR and PPAR-α (all at 1:1000), and anti-β-actin (at 1:5000), all from Proteintech (Proteintech, Chicago, USA) were used in this study. The relative density of protein bands was quantitated with ImageJ version 1.5s (http://imagej.nih.gov/ij/) provided in the public domain by the National Institutes of Health, Bethesda, MD.

### 2.10. Statistical analysis

All values are presented as mean ± SD for each group. The data were analyzed by one-way ANOVA followed by a least significant difference (LSD) post hoc test; the level of significance was set at p < 0.05. SPSS 20.0 software (Chicago, IL, USA) and GraphPad Prism 6.0 software (La Jolla, CA) were used to perform the statistical analysis.

## 3. Results

### 3.1. Female NAFLD patients have significantly lower estrogen and altered gut microbiota compared to the control group

**Fig 1:**
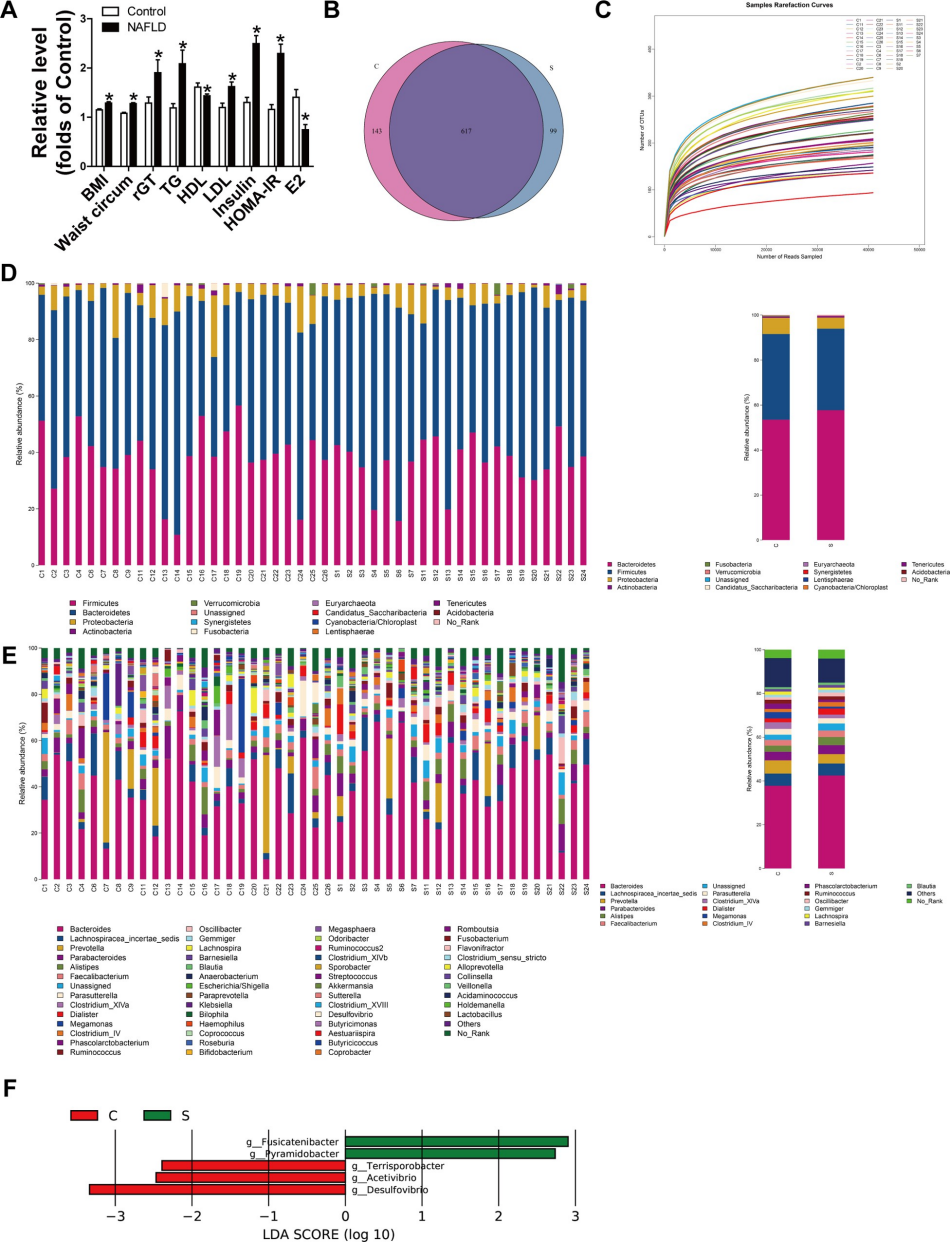
Analysis of clinical baseline characteristics and gut microbiota in NAFLD patients and the control group. **A:** Clinical baseline characteristics showing statistical differences between the two groups. **B:** Venn diagram indicating the differential numbers of OTUs. **C:** Rarefaction curve. **D:** The abundance of bacteria at the phylum level. **E:** The abundance of bacteria at the genus level. **F:** LEfSe analysis shows differentially abundant genera as biomarkers determined by using the Kruskal-Wallis test (P< 0.05) with LDA score>3.5. BMI, body mass index; rGT, ϒ-Glutamyltranspeptidase; TG, triglyceride; HDL, high density lipoprotein; LDL, low density lipoprotein; E2, estradiol; C, the control group (n = 24); S, the NAFLD patients (n = 21). *P < 0.05.

According to the lab design, females were divided into the NAFLD and control group depending on the ultrasound results for fatty liver. General characteristics of the enrolled people have been listed as Table 1. The results showed that there was no significant difference in age between the two groups. Also as shown in Fig 1A, the NAFLD group had a higher BMI and waist circumference, which are characteristics of obesity. However, blood glucose levels showed no statistical difference, while triglyceride, LDL, insulin, and HOMA-IR were higher in the NAFLD group, indicating the NAFLD group were insulin resistant. The average estrogen level of the NAFLD group was lower than that in the control group (74.7 ± 10.3 pg/mL vs 140.6 ± 15.7 pg/mL).

**Table 1:** Characteristics of the enrolled NAFLD patients and control people.

Characteristics	control	NAFLD	Statistics	*P* value
	N = 24	N = 21		
Age (years)	37.6±2.3	38.7±3.2	1.38	0.174
Height (cm)	163.6±3.7	164.2±5.0	0.56	0.579
Weight (kg)	62.1±8.2	67.5±11.0	2.16	0.034
Body mass index (kg/m2)	22.9±2.4	25.8±2.9	4.00	<0.001
Waist Circumference (cm)	75.7±6.5	89.3±8.1	7.11	<0.001
Systolic BP (mmHg)	117.9±12.1	121.0±13.6	0.94	0.349
DiastolicBP (mmHg)	74.6±7.4	74.7±8.4	0.07	0.948
Alanine aminotransferase (U/L)	16.7±9.5	17.2±12.2	0.15	0.878
Aspartate aminotransferase (U/L)	16.2±4.3	15.9±4.7	0.28	0.783
γ-Glutamyl transpeptidase (U/L)	16.7±8.6	24.7±8.9	2.10	0.040
Alkaline phosphatase(U/L)	55.8±10.1	57.8±11.5	0.69	0.494
Total cholesterol (mmol/L)	4.6±0.9	4.8±0.9	0.89	0.376
Triglycerides (mmol/L)	0.9±0.3	1.6±1.2	3.09	0.003
HDL (mmol/L)	1.6±0.4	1.4±0.2	2.03	0.047
LDL (mmol/L)	2.6±0.8	3.2±0.9	2.59	0.011
Fasting glucose (mmol/L)	4.9±0.4	5.1±0.7	0.41	0.683
Fasting Insulin (uIU/ml)	7.8±3.4	14.9±5.3	6.18	<0.001
HOMA-IR	1.7±0.8	3.4±1.4	5.38	<0.001
Estrogen (pg/mL)	140.6±15.7	74.7±10.3	3.50	<0.001

Data are expressed as mean ± SD.

N, number; SD, standard deviation; BP, blood pressure; HDL, high-density lipoprotein; LDL, low-density lipoprotein; HOMA-IR, Homeostatic Model Assessment of Insulin Resistance.

To investigate whether the gut microbiota composition differed between the two groups, we performed Illumina HiSeq 16S rRNA gene sequencing, which produced 8557224 clean reads from 45 samples. The extent of the OTUs (operational taxonomic units) shared between the two groups are summarized in the Venn diagram (Fig 1B). 617 OTUs were common, while 143 were unique to the control and 99 to the NAFLD group, revealing less OTU diversity in the NAFLD group. Rarefaction curves indicated that most gut microbial organisms in each sample were captured with the current sequencing depth (Fig 1C). As illustrated in Fig 1D, *Bacteroidetes*, *Firmicutes*, and *Proteobacteria* were the three dominant phyla. The abundance of *Bacteroidetes* and *Proteobacteria* exhibited significant differences between the two groups. The differences in the genera level are shown in Fig 1E. The abundance of *Bacteroides*, *Alistipes*, and *Unassigned* was much higher in the NAFLD group than control. LEfSe (LDA Effect Size) was used to explore significant changes and relative richness in the bacterial communities of the two groups, and five genera were identified with LDA scores (Fig 1F). Collectively, these result showed that estrogen affected the composition of the gut microbiota.

### 3.2. Estrogen deficient mice develop severe NAFLD, and FMT attenuates NAFLD induced by estrogen deficiency

**Fig 2:**
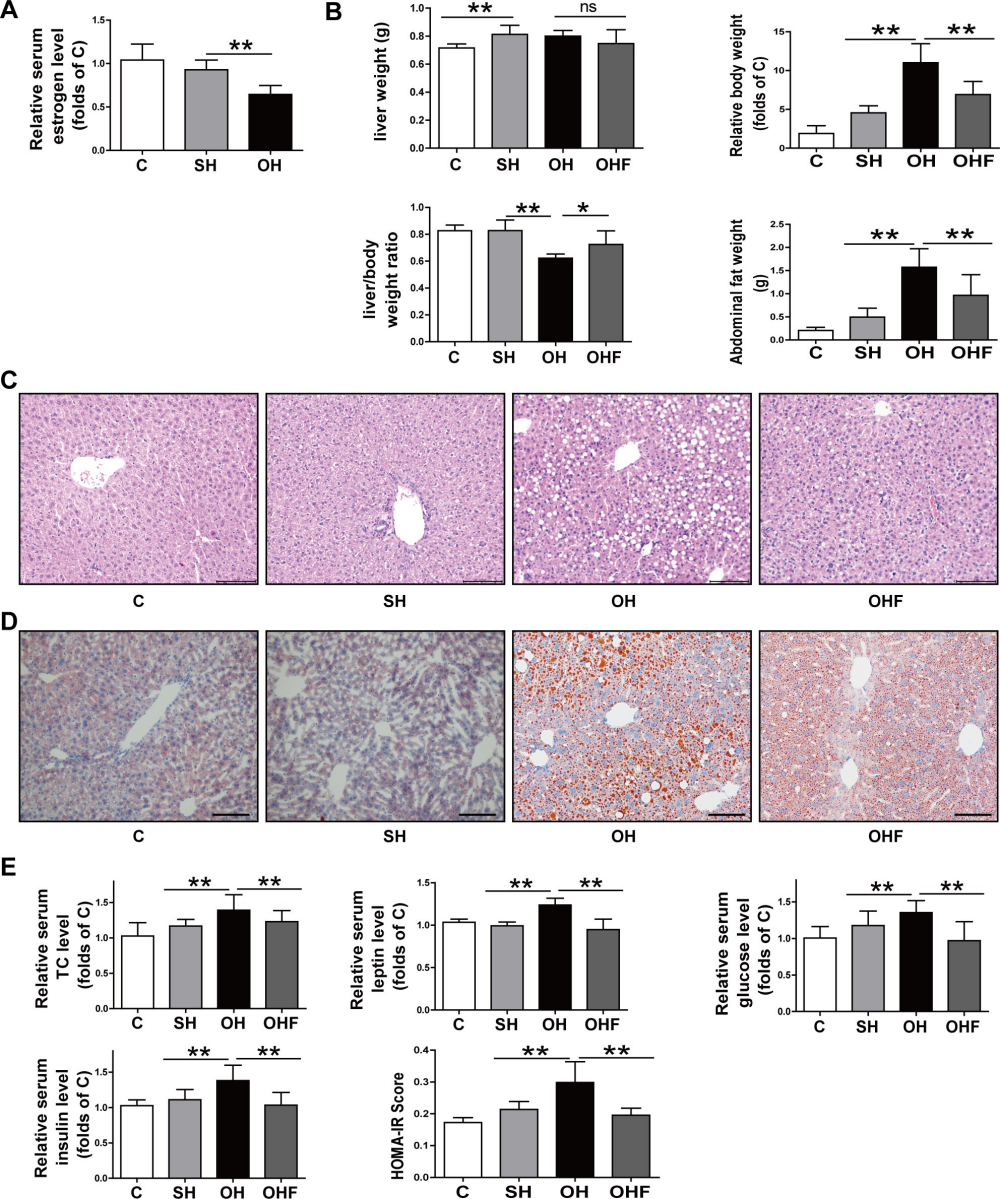
Estrogen deficiency aggravated NAFLD in mice. Interfering with gut microbiota alleviated NAFLD. Tissue and serum were obtained from mice sacrificed at 4 w after ovariectomy and HFD. **A:** Comparison of estrogen level among the C, SH and OH groups. **B:** The liver weight, body weight, liver/body weight ratio and abdominal fat weight among the four groups. **C:** Liver tissues stained with hematoxylin and eosin. **D:** Liver tissues stained with Oil Red O. **E:** Serum TC, leptin, glucose, insulin level and HOMA-IR. C, normal diet (n = 8); SH, sham+HFD (n = 10); OH, OVX+HFD (n = 8); OHF, OVX+HFD+FMT (n = 10); OVX, ovariectomy; HFD, high fat diet; FMT, fecal transplantation; TC, total cholesterol. *P < 0.05; **P < 0.01.

To investigate whether estrogen deficiency is a promoter of NAFLD, mice were administrated with OVX followed by an HFD to induce NAFLD. At the same time, FMT was administered to some of the OH mice to observe whether FMT would attenuate the NAFLD (OHF group). As shown in Fig 2A, following OVX, the OH group had lower levels of estrogen than the SH group, indicating that OVX successfully reduces estrogen in animal models. Fig 2B shows liver weight was increased after mice were treated with HFD, but liver weight in SH, OH and OHF groups showed no differences. Body weight was obviously increased in OH mice but was much lower in SH and OHF groups. Contrarily, liver/body weight ratio was decreased in OH mice. In addition, an increase in abdominal fat was found in the OH group, however, the OHF group had significantly less.

According to the HE staining, the OH group showed more hepatic steatosis than the SH group, in that it had more lipid droplets and inflammatory cell infiltration. But the OHF group showed greater mitigation in hepatic steatosis than the OH group (Fig 2C). The Oil Red O staining results showed that lipid droplets were further enhanced in OH mice compared with the SH mice, yet lipid accumulation within the liver tissues were alleviated in OHF mice (Fig 2D). These results indicated that estrogen reduction would exacerbate NAFLD, but that FMT could inhibit the NAFLD in the OH mice. In addition, the NAFLD condition was assessed by the serum biochemical index. Compared with the OH group, the SH and OHF groups both had lower total cholesterol (TC) and leptin than the OH group. Serum glucose, insulin and HOMA-IR level in OH group were higher than in the SH and OHF group (Fig 2E). Although the amounts of ALT, AST and triglyceride (TG) in the OH group were slightly higher than in the SH group, no statistical differences were found. Put all together, these results indicated that estrogen reduction worsened NAFLD, while FMT could significantly relieve the condition.

### 3.3. The composition of the gut microbiota was changed in mice with NAFLD induced by OVX

**Fig 3:**
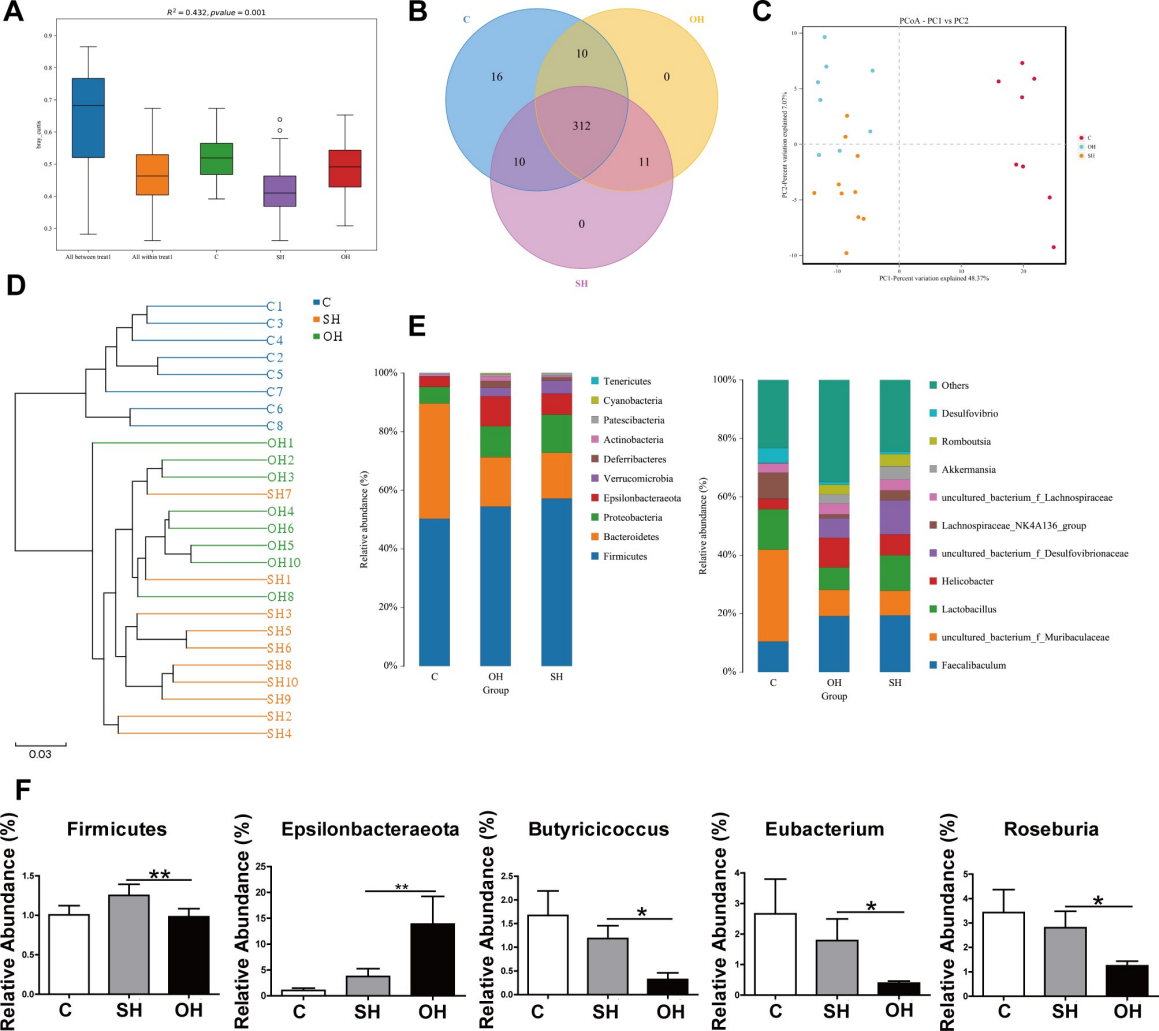
Estrogen reduction changed the structure of gut microbiota in OH model mice. **A:** Shannon index calculated after rarefying to an equal number of sequence reads for all samples. The Shannon indexes were decreased after OVX and HFD treatments. **B:** Venn diagram indicating the differential numbers of OTUs in each group. **C:** PCoA score based on weighted Unifrac metrics differed in each group. **D:** System clustering tree of gut microbiota based on weighted. Unifrac metrics indicated the different beta diversity of gut microbiota in each group. **E:** OVX and HFD treatment changed the microbial community at the phylum and genus levels. **F:** The relative abundance of *Firmicutes*, *Butyricicoccus*, *Eubacterium* and *Roseburia* was decreased, whereas *Epsilonbacteraeota* was increased in the OH group compared with the SH group. C, normal diet (n = 8); SH, sham+HFD (n = 10); OH, OVX+HFD (n = 8), OVX, ovariectomy; HFD, high fat diet. *P < 0.05; **P < 0.01.

We then investigated whether OVX results in alterations in the composition of the gut microbiota. In total, 2,164,126 useable reads and 359 OTUs were obtained from 26 samples. The Shannon indexes were lower in the SH group compared with C groups (Fig 3A). Moreover, a Venn diagram of the three groups revealed that 312 OTUs overlapped among the groups: 322 OTUs were present in both the C and SH groups; 322 in both the C and OH groups; and 323 in both the SH and OH groups (Fig 3B). PCoA (principal co-ordinates analysis) showed that gut microbiota in the C group were different from those in the SH and OH groups (Fig 3C). The system clustering tree also indicated significant differences between each group, although the distance between the SH group and the OH group was small (Fig 3D).

We further investigated the gut microbiota species and their relative abundance. As shown in Fig 3E, at the phylum level, 10 phyla could be found in all samples and the most abundant phyla in all samples were: *Firmicutes*, *Bacteroidetes*, *Proteobacteria*, and *Epsilonbacteraeota*. Conversely, *Verrucomicrobia* were detected in the SH and OH groups but not in the C group. Furthermore, at a genus level classification, *Faecalibaculum* and *Helicobacter* were more common in the SH and OH groups but less abundant in the C group. Interestingly, results from Fig 3E show that the presence of *Muribaculaceae* and *Lactobacillus* in the SH and OH groups were significantly decreased relative to those in the C group. Furthermore, the presence of *Firmicutes* was significantly lower in the OH group compared with that in the SH group; whereas the relative abundance of *Epsilonbacteraeota* was remarkably higher. The relative abundance of *Butyricicoccus*, *Eubacterium*, and *Roseburia* were diminished in the OH group compared with the SH group (Fig 3F).

**Fig 4:**
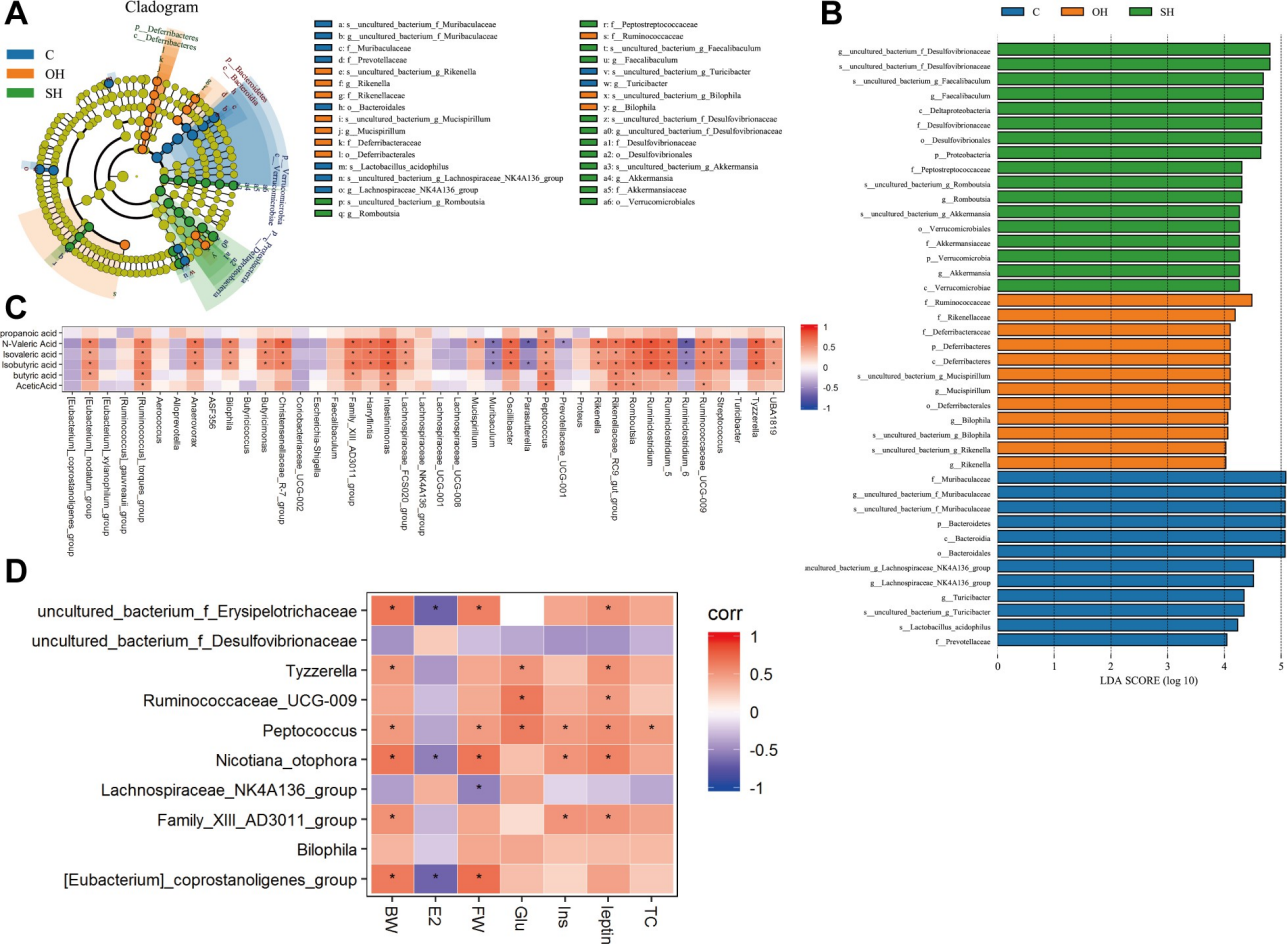
Analysis of gut microbiota in mice. **A:** Cladogram of gut microbiota in each group. **B:** LEfSe analysis shows differentially abundant genera as biomarkers determined using the Kruskal-Wallis test (P<0.05) with LDA score>3.5. **C, D:** Correlation between the gut bacterial genera and SCFA (C) or serum indicator (D). C, normal diet (n = 8); SH, sham+HFD (n = 10); OH, OVX+HFD (n = 8); OVX, ovariectomy; HFD, high fat diet; BW, body weight; E2, estradiol; FW, abdominal fat weight; Glu, glucose; Ins, insulin; TC, total cholesterol.

LefSe analysis was performed with the pooled data to identify specific taxa that could be used as biomarkers and dominant microbiota for each group. A cladogram for family and genus level abundance is shown in Fig 4A. In total, 41 genera were identified with LDA scores > 3.5 (Fig 4B). For correlation analysis, genera level correlations with SCFA and serological indicators such as Estrogen, TC, and leptin are shown in Fig 4C and 4D. *Peptococcus* and *Romboutsia* were positively, while *Ruminiclostridiun-6* and *Muribaculum* were negatively correlated with SCFA. The occurrence of the genera exhibited a positive correlation with TC, leptin and body weight. Surprisingly, estrogen showed negative correlations with most of the genera detected. Put all together, our results showed that estrogen could modulate gut microbiota composition in NAFLD model mice.

### 3.4. Butyrate is decreased in NAFLD patients and in the OH mice, and supplementation with butyrate inhibits NAFLD caused by estrogen reduction

**Fig 5:**
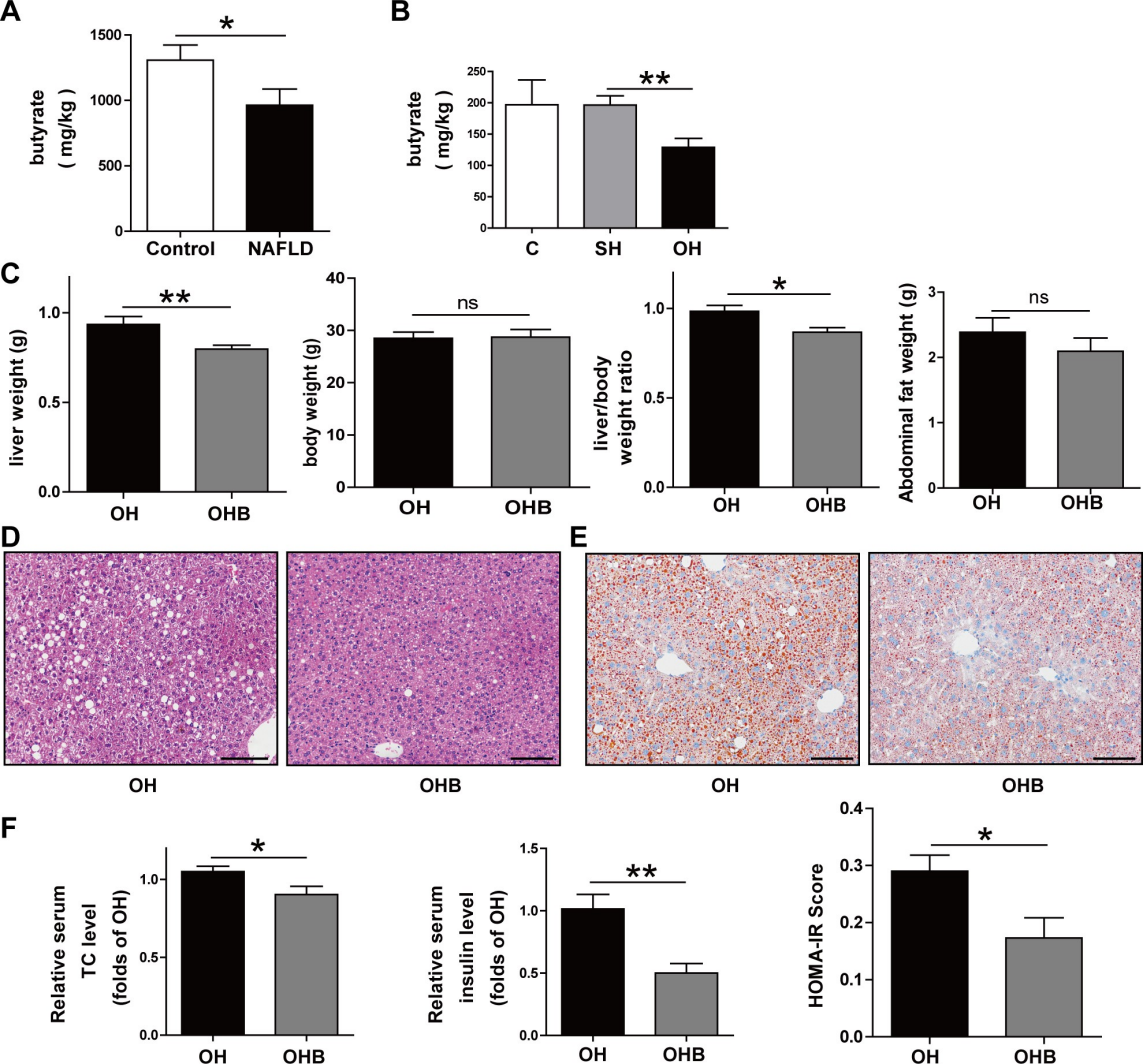
Butyrate was decreased in the NAFLD patients and OH mice. Supplement with butyrate eased NAFLD. After 4 w of ovariectomy and high fat diet, the OH mice received another 4 w of butyrate feeding (named OHB). **A:** Butyrate contents in the NAFLD patient with decreased estrogen. **B:** Butyrate contents in the C, SH and OH mice groups. **C:** Comparison of liver weight, body weight, liver/body weight ratio and abdominal fat weight between OH and OHB groups. **D:** Liver tissues stained with hematoxylin and eosin. **E:** Liver tissues stained with Oil Red O. **F:**Serum TC, insulin and HOMA-IR level. C, normal diet (n = 8); SH, sham+HFD (n = 10); OH, OVX+HFD (n = 8); OHB, OVX+HFD+butyrate (n = 10); OVX, ovariectomy; HFD, high fat diet; TC, total cholesterol. *P < 0.05; **P < 0.01.

To determine whether SCFA was changed in NAFLD patients with decreased estrogen, the concentration of their SCFA was examined. The butyrate content was much lower in the NAFLD patients than that in the controls (Fig 5A). However, the concentration of other SCFAs showed no difference between the two groups. Butyrate was also significantly decreased in the OH mice compared with the SH group (Fig 5B). Consistently, the other SCFAs showed no change. The content profile of SCFAs in OH mice was similar to that found in the NAFLD patients with decreased estrogen. These results indicate that the butyrate content is affected by estrogen in the NAFLD model.

To determine whether decreased butyrate causes the NAFLD according to estrogen reduction, the OH group was given butyrate (200mg/kg) for 4 weeks (named the OHB group). Results showed that the OHB group had no statistical difference in body and abdominal fat mass compared with the OH group, but compared with the OH mice, liver weight and liver/body weight ratio were clearly decreased in OHB mice (Fig 5C). HE staining also demonstrated that hepatocyte steatosis was reduced in the OHB group (Fig 5D). More importantly, the Oil Red O staining showed mice feeding with butyrate were apparently associated with attenuation of hepatic lipid deposition (Fig 5E), Furthermore, serum biochemical indicators also showed lower serum TC, insulin and HOMA-IR in OHB mice, but no statistical differences were shown between the groups for ALT and AST (Fig 5F).

### 3.5. Estrogen reduced mice have lower production of antimicrobial peptide (AMP) in IEC

**Fig 6:**
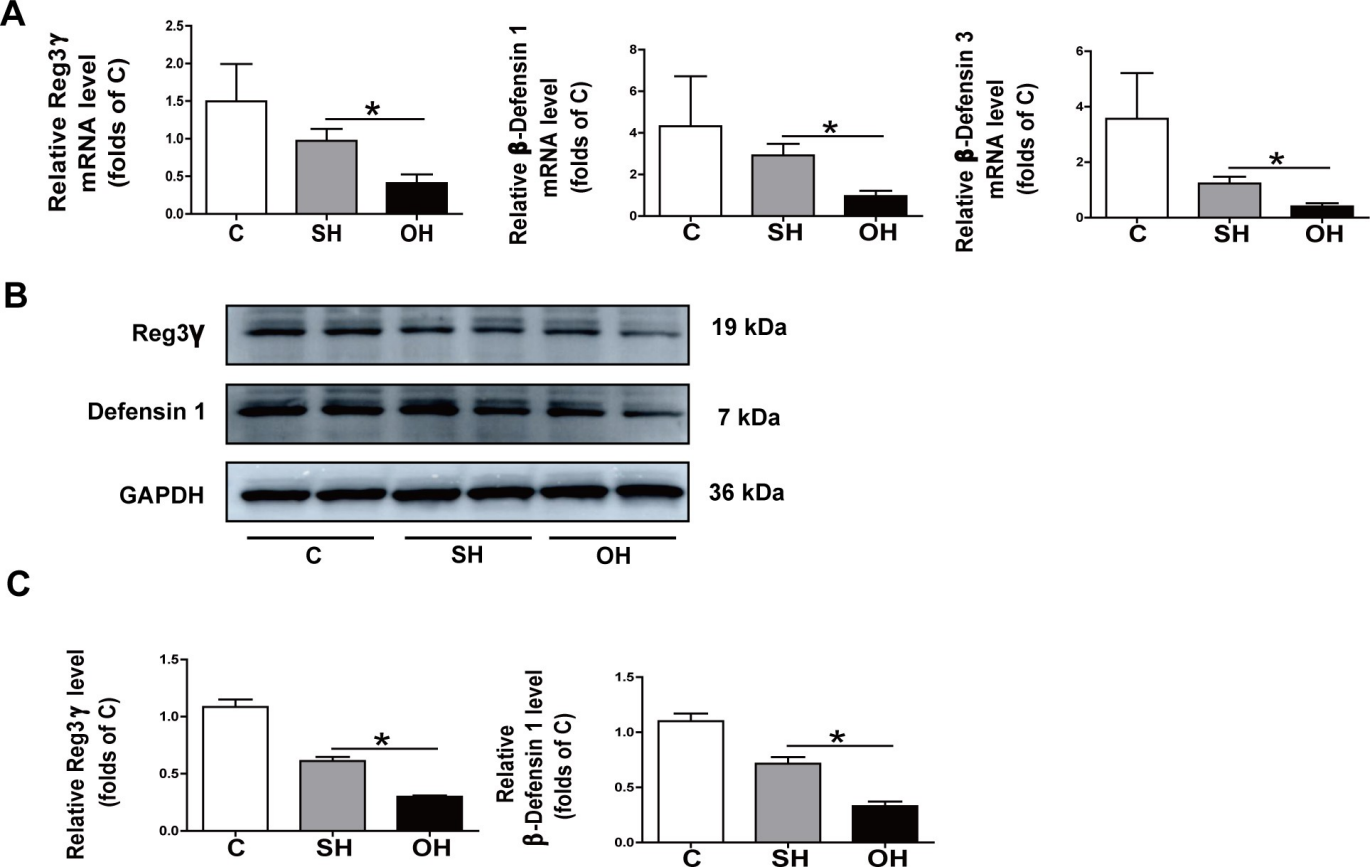
Decreased production of Reg3γ and β-defensins in IEC of OH mice. IEC were isolated from the small intestines of C, SH and OH mice, respectively. **A:** IEC expression of Reg3γ and β-defensins 1 and 3 in OH and the control mice were determined by qRT-PCR. **B:** Protein levels of Reg3γ and β-defensins 1 were determined by western blot. **C:** Relative density ratio of Reg3γ and β-defensins 1. IEC, intestinal epithelial cell; qRT-PCR, quantitative real-time polymerase chain reaction; C, normal diet (n = 8); SH, sham+HFD (n = 10); OH, OVX+HFD (n = 8); OVX, ovariectomy; HFD, high fat diet. *P < 0.05.

We then investigated whether the altered microbiota and butyrate levels in estrogen reduced mice affect IEC function. AMP produced by IEC cells play a critical role in regulation of host and microbiota interaction. In order to study AMP production, IEC were collected to determine their AMP expression. Based on the results from qRT-PCR, the OH group had decreased expression of Reg3γ, β-defensins 1 and 3 than the SH group at mRNA levels (Fig 6A), and protein level of Reg3γ and β-defensins 1 were also decreased (Fig 6B and 6C). However, no difference was detected between the two groups for β-defensins 4. These results confirmed that AMP production was decreased in mice with NAFLD caused by estrogen reduction.

### 3.6. Expression of intestinal epithelial tight junction related genes and proteins are changed in estrogen reduced mice

**Fig 7:**
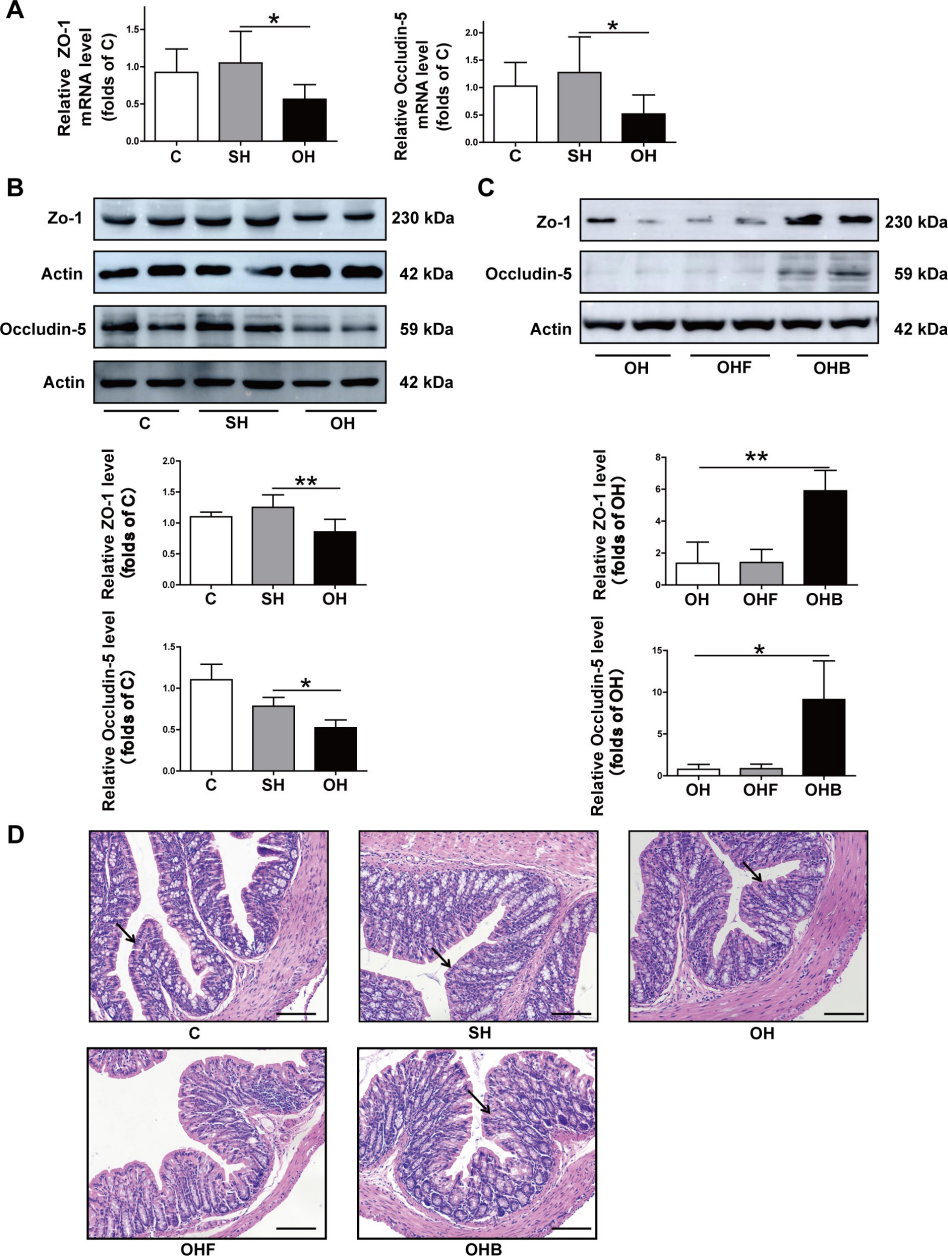
Analysis of the activity of tight junction protein in the small intestine in the mice. **A:** RT-qPCR analysis of the mRNA level of ZO-1 and Occludin-5. **B:** Western blots and density ratios of ZO-1 and Occludin-5. **C:** Representative histological staining (H&E) of small intestine tissue. C, normal diet (n = 8); SH, sham+HFD (n = 10); OH, OVX+HFD (n = 8); OHF, OVX+HFD+FMT (n = 10); OHB, OVX+HFD+butyrate (n = 10); OVX, ovariectomy; HFD, high fat diet. *P < 0.05; **P < 0.01.

The mechanism by which hepatic steatosis induced by estrogen reduction was studied. Because the integrity of the intestinal epithelium plays a crucial role in maintaining a balance in fatty acid intake, damage to the intestinal epithelial barrier leads to an increase in fatty acid intake and potentially to fatty liver disease. The barrier function of the intestinal epithelium were investigated. The expression of the ZO-1 and Occludin-5 genes were lower in the OH group than in the SH group (Fig 7A), and expression of the protein ZO-1 and Occludin-5 had significantly declined in the OH group (Fig 7B). In addition, compared with the OH mice, ZO-1 and Occludin-5 were clearly increased in butyrate feeding mice but not changed in OHF mice (Fig 7C). Likewise, compared with the SH mice, HE staining showed that the OH and OHF group had reduced number of epithelium cells nucleus, with thickening of the intestinal wall. However, butyrate treatment mice seemed to block the reduction of intestinal epithelium cells (Fig 7D). The results confirmed that the intestinal epithelial barrier function was damaged in OH mice, but was relieved when mice were treated with butyrate.

### 3.7. Fatty acid (FA) synthesis, intake, and oxidation related gene and protein expression are changed in estrogen reduced mice

**Fig 8:**
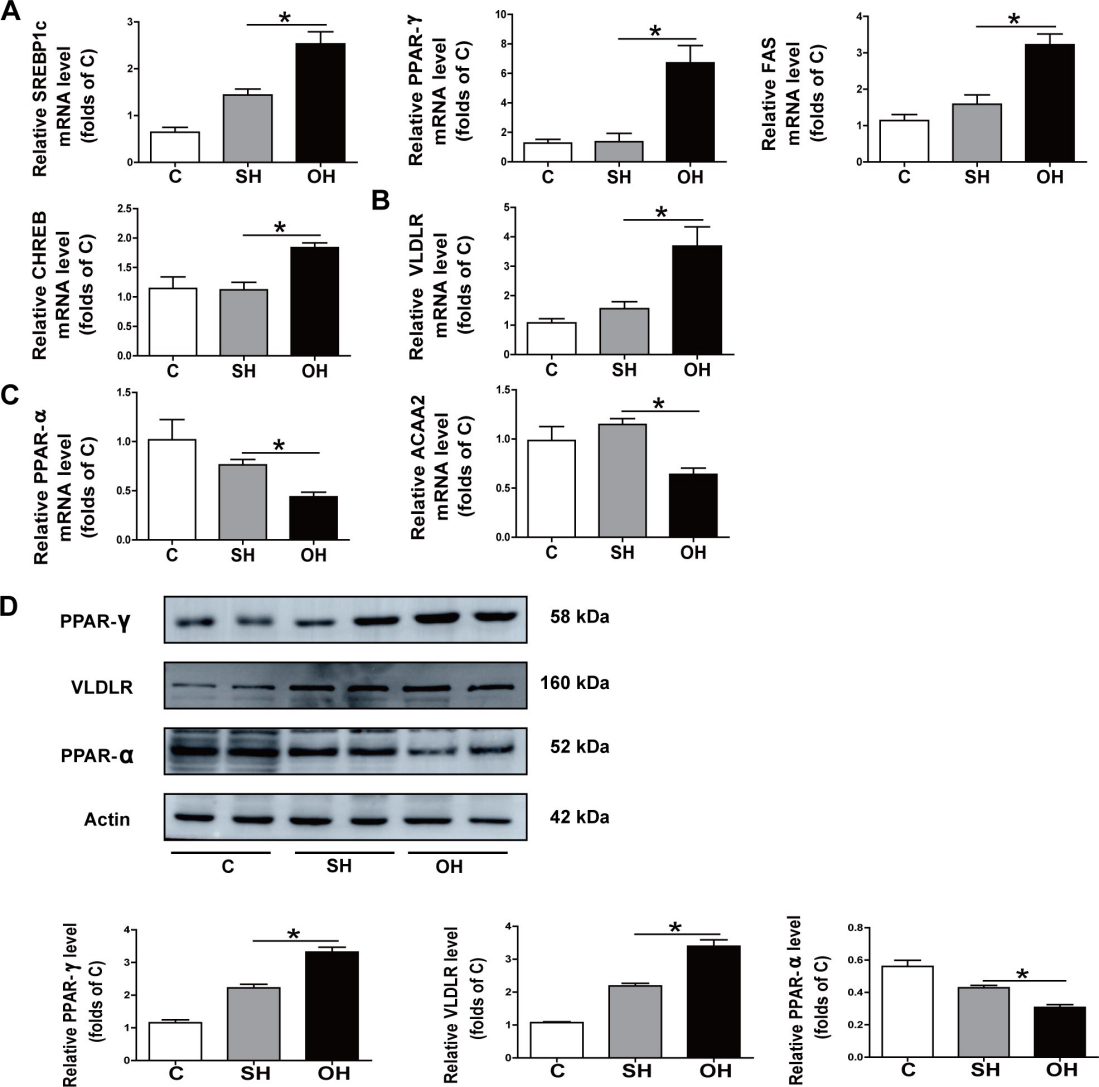
Expression of fatty acid synthesis, intake and oxidation related genes and proteins in the mice. **A:** Analysis with qRT-PCR on the expression of SREBP1, PPAR-ɣ, FAS and CHREB in the three groups. **B:** Analysis with qRT-PCR on the expression of VLDLR in the three groups. **C:** Analysis with qRT-PCR on the expression of PPAR-ɑ and ACAA2 in the three groups. **D:** Western-assisted analysis and relative density ratio of PPAR-ɣ, VLDLR, PPAR-ɑ. C, normal diet (n = 8); SH, sham+HFD (n = 10); OH, OVX+HFD (n = 8); OVX, ovariectomy; HFD, high fat diet. *P < 0.05.

As the central organ in the metabolic reactions, the liver plays an important role in maintaining metabolic balance. NAFLD is a disease expressed through multiple metabolic disorders and especially in FA metabolism. We hypothesize that mice with an estrogen reduction have a disorder of FA metabolism pathway. Therefore, genes and protein related to FA metabolism were tested in these mice. The expression of the lipid synthesis-related genes, SREBP1, PPAR-ɣ, FAS and CHREB were found to be significantly increased with estrogen reduction (Fig 8A). Moreover, in comparison with the SH group mice, the OH group had a significantly greater expression of the lipid intake related gene VLDLR (Fig 8B), while the lipid oxidation related genes, PPAR-ɑ and ACAA, had lower expression in the OH group (Fig 8C). The Western blot shown that the OH group mice had increases in PPAR-ɣ and VLDLR proteins but PPAR-ɑ expression had decreased (Fig 8D). In summary, estrogen was shown to be a crucial factor in FA metabolism regulation in the liver.

## 4. Discussion

NAFLD is a frequent cause of chronic liver disease, but the therapeutic options for this disease are limited. Although the reasons for the increased incidence of obesity and NAFLD in postmenopausal women are not fully understood, the reduction in estrogen levels is considered an important obesity-triggering factor. Previous studies have shown the promotional effect of estrogen deficiency during the process of hepatic steatosis in menopausal women [4]. Moreover, with the decrease in estrogen, OVX mice tend to accumulate body fat and become obese [19]. Several studies have shown that targeted intervention with estrogen is therapeutically efficient in inhibiting NAFLD, including a study by Wagner [20]. In current study, we found that women aged 35–45 with NAFLD had significantly lower estrogen levels than the controls, while they also had higher BMI, waist circumference, insulin and triglyceride levels, indicating that decreased estrogen is a factor that promotes NALFD. Additionally, in the animal model, OVX resulted in serious hepatic steatosis, while FMT markedly alleviated NAFLD in OVX mice. All these results suggest that, although the pathophysiological role of estrogen deficiency in hepatic steatosis has not been fully elucidated, it is undoubtedly a prominent feature.

Growing evidence supports the role of gut microbiota in obesity and NAFLD. Indeed, accumulating evidence indicates that compositional changes in gut microbiota have been proposed as mechanistically contributing to the progression of NAFLD [21–23]. A study by Boursier et al. [24] demonstrated that the severity of NAFLD is associated with gut dysbiosis and a shift in the metabolic function of the gut microbiota. Moreover, Rabot et al. [25] observed that GF mice receiving HFD showed enhanced insulin sensitivity with improved glucose tolerance and reduced insulinaemia. This is in line with the study of Kreznar et al. [26]. In our study, we also found that the structure of the gut microbiota was changed in NAFLD patients and mice induced by OVX. Additionally, our data also demonstrated that the expression of AMP was inhibited in OVX mice with decreased butyrate content, which is in line with the study by Cong et al. [27]. In light of this, drugs targeting AMP production through the promotion of intestinal homeostasis might serve as promising candidates for clinical application. Gut microbiota-targeted interventions may prove to be an effective and innovative approach for treating NAFLD. FMT has been shown to be effective in the treatment of severe gastrointestinal diseases [28]. Le Roy et al. [7] showed that susceptibility to the development of NAFLD was transmissible via FMT between mice, indicating a potential causative role for gut microbial dysbiosis in the occurrence or evolution of the hepatic disease. Consistent with these results, our study also demonstrated that FMT can markedly attenuate NAFLD caused by estrogen deficiency in an animal model. Our study also provides the evidence for the effective treatment of NAFLD in menopausal women. It makes sense to explore possible therapeutic interventions targeting the gut microbiota.

Many studies have shown abnormal levels of SCFA in NAFLD patients [24, 29]. Through reabsorption and distribution, SCFA can affect intestinal barrier health, which is very important in the pathogenesis of NAFLD. Destruction of the gut barrier has been associated with the translocation of lipopolysaccharides (LPS), leading to endotoxemia that drives NAFLD progression [30]. SCFAs, mainly butyrate and propionate, reduce gut inflammation and preserve gut barrier integrity, potentially limiting the translocation of LPS [31]. Consistent with these results, our study also demonstrated that butyrate is significantly decreased in the NAFLD patient and mice with decreased estrogen, while supplementing with butyrate eased hepatic steatosis, indicating butyrate as a potential therapy for NAFLD in menopausal women.

Increased uptake of circulating FA, increased hepatic lipogenesis, reduced rate of FA oxidation and reduced FA secretion are the multiple mechanisms that lead to an increased accumulation of lipids in the liver [32]. Studies have shown that butyrate restores PPARα activation in HFD fed rats, thus enhancing FA β-oxidation, inhibiting lipid synthesis and downregulating nuclear factor-kappa B pathways and inflammation [33, 34]. In the current report, our data also showed that OVX mice with decreased butyrate content had upregulation of FA intake and synthesis but downregulation of oxidation related gene and protein expression. These observations clearly emphasize the importance of the lipid metabolism balance in the development of hepatic steatosis.

In conclusion, our findings provide evidence that estrogen deficiency deteriorates NAFLD, interfering with gut bacteria and supplementing with butyrate can markedly attenuate NAFLD. By identifying the mechanism by which estrogen regulates NAFLD, our findings provide a rationale for novel therapeutic approaches to ameliorate NAFLD. Further studies are needed to elucidate the interaction of estrogen with gut microbiota in greater detail, with the aim of identifying targets for pharmacological intervention to mimic the protective effects of estrogen. Due to the severe side effects of estrogen, the closer investigation into manipulating gut bacteria as a strategy for the pharmacological amelioration of NAFLD appears a promising goal. Nevertheless, although estrogen has been demonstrated to protect the liver from steatosis, the characterization of its role against NAFLD via the gut bacteria and butyrate will expand our understanding of this liver protector through new insights.

## 5. Conclusions

In this study, we demonstrated that estrogen deficiency promotes and accelerates the deterioration in NAFLD. The composition of gut microbiota in both NAFLD patients and mice was altered, and FMT attenuated estrogen deficiency induced NAFLD in mice. Butyrate, a metabolite of the gut microbiota, was significantly decreased in both NAFLD patients and OVX mice. Supplementing with butyrate improved the NAFLD condition of the mice. These results highlight the importance of the gut microbiota and its metabolite SCFA in regulation of NAFLD caused by estrogen deficiency. To the best of our knowledge, this study innovatively documents the key role of the gut microbiota and SCFA in a NAFLD model induced by estrogen deficiency.

## Supporting information

S1 Checklist STROBE statement—checklist of items that should be included in reports of observational studies. (DOCX)

S1 Raw images (PDF)

## Abbreviations

NAFLD: nonalcoholic fatty liver disease
SCFA: short chain fatty acids
OVX: ovariectomy
FMT: fecal bacteria transplantation
AMP: antimicrobial peptides
NASH: non-alcoholic steatohepatitis
HCC: hepatocellular carcinoma
HFD: high fat diet
C: normal diet group
SH: sham-operated+HFD group
OH: ovariectomy+HFD group
OHF: ovariectomy+HFD+fecal transplantation group
OHB: ovariectomy + HFD+butyrate group
ALT: alanine aminotransferase
AST: aspartate aminotransferase
IEC: intestinal epithelial cells
BMI: body mass index
OTUs: operational taxonomic units
LPS: lipopolysaccharides
FA: fatty acids
