# Supplementary figures and images for: Gut microbiota and butyrate contribute to nonalcoholic fatty liver disease in premenopause due to estrogen deficiency
Source: PLoS One. 2022 Feb 2;17(2):e0262855. doi: 10.1371/journal.pone.0262855 (PMC8809533; doi:10.1371/journal.pone.0262855)

Fig. 6B

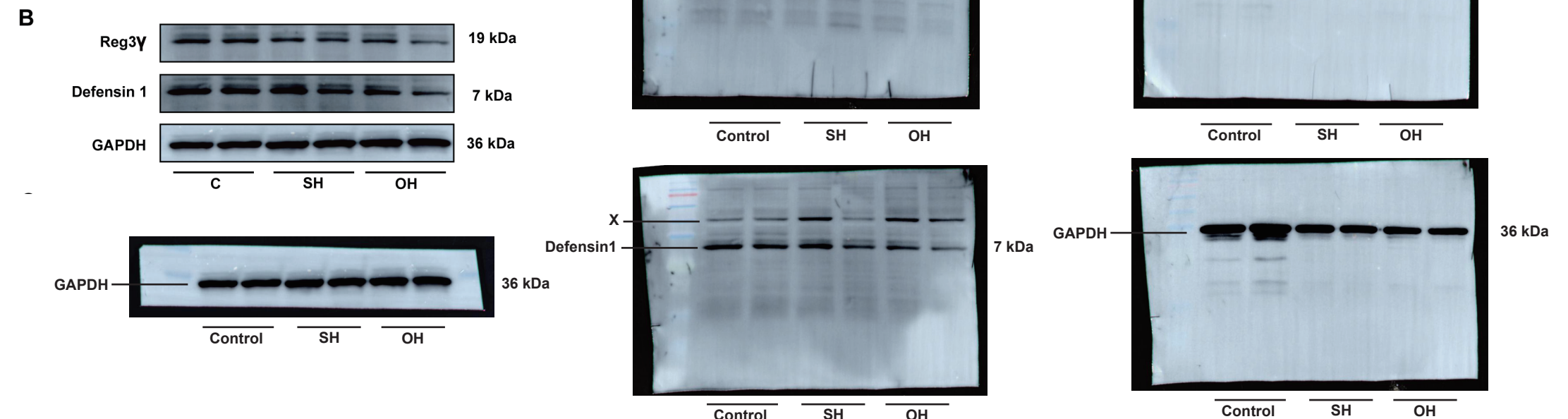

Fig. 7B

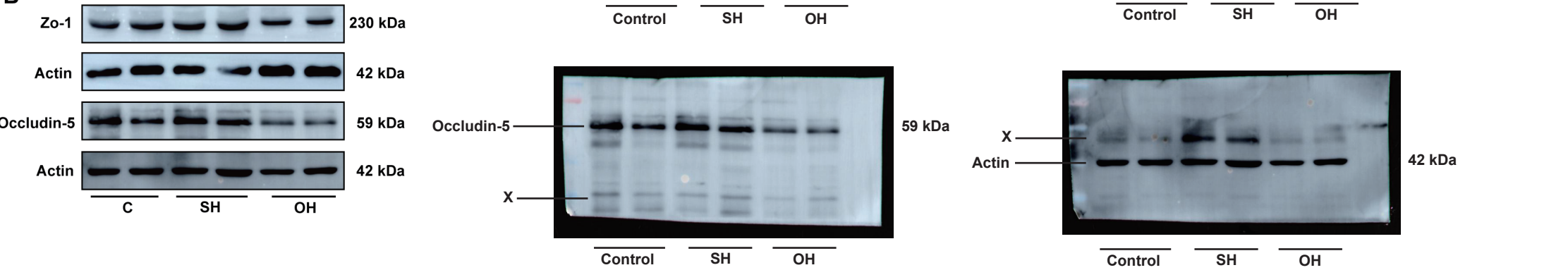

Fig. 7C

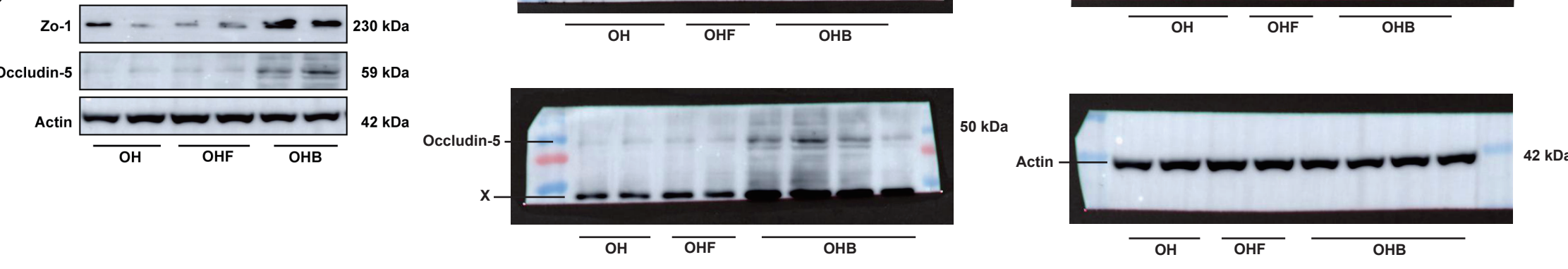

Fig. 8D

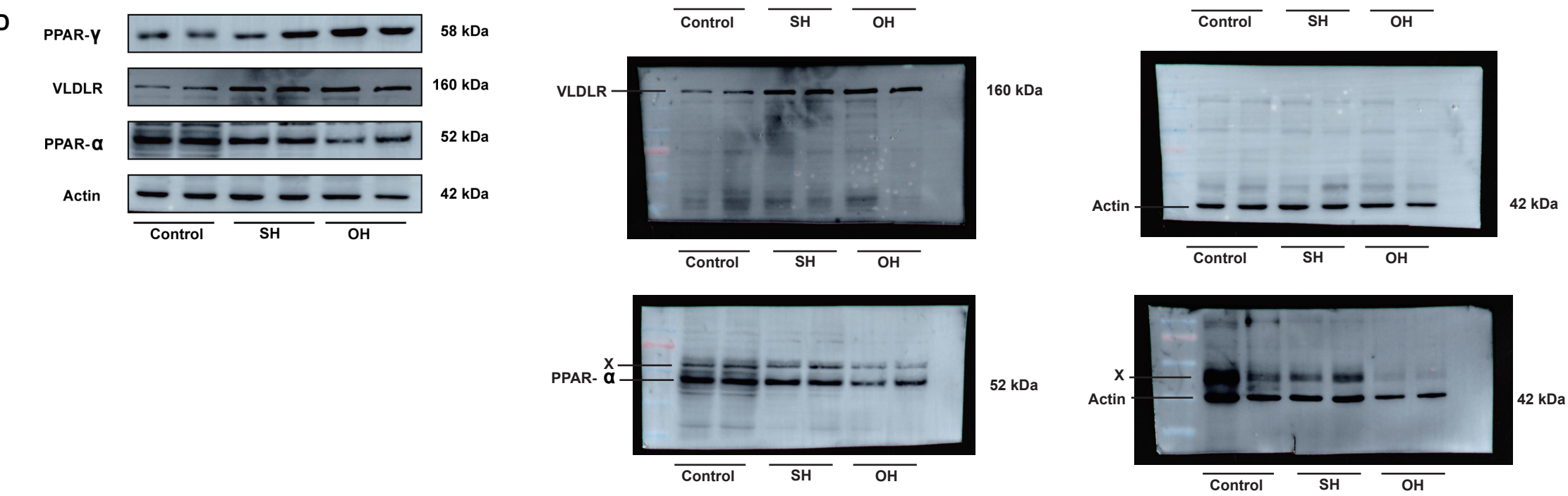

Supplement: S1 Raw images — (PDF) [file pone.0262855.s002.pdf]
